# Efficient 1,4-addition of α-substituted fluoro(phenylsulfonyl)methane derivatives to α,β-unsaturated compounds

**DOI:** 10.3762/bjoc.4.17

**Published:** 2008-05-21

**Authors:** G K Surya Prakash, Xiaoming Zhao, Sujith Chacko, Fang Wang, Habiba Vaghoo, George A Olah

**Affiliations:** 1 Loker Hydrocarbon Research Institute and Department of Chemistry, University of Southern California, Los Angeles, California 90089-1661

## Abstract

The 1,4-addition of a monofluoromethyl nucleophile to a variety of α,β-unsaturated compounds has been achieved under mild conditions using either phosphines or potassium carbonate at room temperature. α-Substituted fluoro(phenylsulfonyl)methane easily undergoes Michael addition to α,β-unsaturated ketones, esters, nitriles, sulfones, as well as propynoates at room temperature to yield the corresponding adducts in moderate to excellent yields.

## Background

Compounds with a monofluoromethyl moiety are of great importance with regards to isostere-based drug design [1–4]. Consequently, synthesis of new functionalized α-monofluorine-substituted active methylene derivatives has attracted considerable attention particularly in the field of medicinal chemistry [5–6]. One of the major interests in our group has focused on developing new fluorination reagents or fluorinated building blocks for preparation of fluorine-substituted compounds [7–13]. As part of our ongoing effort to extend the applications of fluorine-containing (phenylsulfonyl)methane derivatives, we envisaged that fluoro(phenylsulfonyl)methane, α-substituted by nitro, cyano, ester, or acetyl would be useful for the synthesis of functionalized monofluoromethylated compounds, which would undergo various transformations. New synthetic methods for the synthesis of α-substituted fluoro(phenylsulfonyl)methane derivatives under mild reaction conditions, using convenient starting materials, are still desirable.

Fluorinated carbanions are in principle "hard" nucleophiles that readily undergo 1,2-addition with Michael type acceptors instead of 1,4-addition [14–16]. Different strategies have been employed to achieve 1,4-addition, which is still percieved to be a challenge. For instance, Yamamoto [17] and Röschenthaler [18–19] have made use of bulky aluminum Lewis acids to protect the carbonyl of Michael acceptors and thus sucessfully transferred the trifluoromethyl anion generated from the "Ruppert-Prakash reagent" (TMS-CF_3_) in a 1,4-manner rather than the favored 1,2-addition. Portella et al. [20] have shown that 1,4-addition of difluoroenoxysilanes to enones can be used to introduce difluoromethylene moiety while Kumadaki and coworkers [21–22] have used bromodifluoroacetate with a copper catalyst to introduce the CF_2_ functionality. There also exist few reports on the 1,4-addition of monofluoromethylene moieties to α,β-unsaturated compounds [23–24]. Takeuchi and coworkers [25] have shown that α-fluoronitroalkanes can undergo 1,4-addition to methyl vinyl ketone and acrylonitrile to afford the dialkylated products.

## Results and Discussion

Herein, we disclose the facile reaction of fluoro(phenylsulfonyl)methane derivatives and various Michael acceptors. We first began with the preparation of nitro, cyano, ester, or acetyl-substituted (phenylsulfonyl)methanes from the corresponding (phenylthio)methane derivatives, the precursors of α-substituted fluoro(phenylsulfonyl)methane derivatives. Oxidation of (nitromethyl)(phenyl)sulfide with aqueous hydrogen peroxide [H_2_O_2_, 30% (wt)] was attempted in acetic acid at room temperature. Tuning the conditions by using 4-fold excess of H_2_O_2 _afforded 90% yield of (nitromethylsulfonyl)benzene overnight (Table 1, entry 1) [26–28]. **2a**–**c** and **2e** were prepared in 76–91% yields under the optimized condition and used without further purification [30–34]. Interestingly, the oxidation of **1d** gave a mixture of **2d** and methylsulfonylbenzene in a ratio of 2:1. Hence, compound **2d** was synthesized from another known procedure [29]. Fluorobis(phenylsulfonyl)methane **3f** was prepared following a literature procedure by fluorinating bis(phenylsulfonyl)methane, which is commercially available [11].

**Table 1:** Oxidation of sulfides and monofluorination of sulfones.

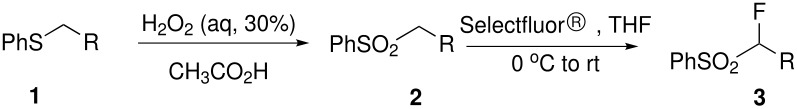					
Entry	R	**2**	Yield^a^ (%)	**3**	Yield^a^ (%)

1	NO_2_	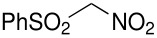 **2a**	90	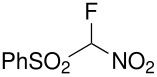 **3a**	62
2	CN	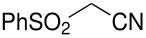 **2b**	76	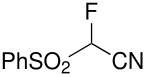 **3b**	45
3	CO_2_Et	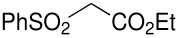 **2c**	83	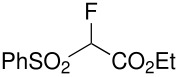 **3c**	56
4	COMe	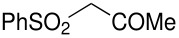 **2d**	^b^	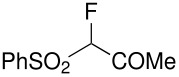 **3d**	61
5	CH=CH_2_	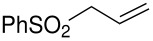 **2e**	91	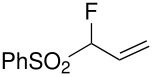 **3e**	^c^

^a^isolated yield, ^b^**2d** was prepared according to ref. [29], ^c^no product obtained

Our strategy for the preparation of monofluoro methanes was to use commercially available Selectfluor^®^ [35] as the electrophilic fluorine source. The monofluorination of (nitromethylsulfonyl)benzene with Selectfluor^®^ under the improved conditions [36] [treatment of (nitromethylsulfonyl)benzene (6.75 mmol) with NaH (6.75 mmol) in THF (25 mL) followed by Selectfluor^®^ (6.75 mmol) in 15 mL of DMF at 0 °C] gave [fluoro(nitro)methylsulfonyl]benzene **3a** in 62% isolated yield (Table 1, entry 1). A doublet was observed at δ −142.16 ppm in the ^19^F NMR spectrum of **3a**, which matched our previously reported result [11]. Other fluoro(phenylsulfonyl)methane derivatives were synthesized in 45–61% yields under similar conditions (Table 1, entries 2–5).

Alkylation of **3a**–**d**, **3f** is reported to be a challenge because the combined carbanion-stabilizing abilities of the two strong electron-withdrawing groups are not sufficient to overcome the well-known carbanion destabilization by the adjacent fluorine [25]. With this in mind, we first opted to use phosphines as nucleophilic catalysts. Concerning applications of **3a**–**d**, and **3f** in constructing the carbon-carbon bond, a reaction of [fluoro(nitro)methylsulfonyl]benzene with methyl vinyl ketone was first tested in the presence of PPh_3_ (50 mol%) in THF at room temperature under argon. Interestingly, 5-fluoro-5-nitro-5-(phenylsulfonyl)pentan-2-one (**5a**) was obtained in 93% yield as the sole product, which was characterized by ^1^H, ^13^C, ^19^F NMR, and HRMS. In the ^19^F NMR spectrum of **5a**, two doublets at δ −125.94 ppm were observed. As it is known, fluoro substitution can cause problems in transformations, since fluoride can also act as a leaving group. Notably, fluorine-free products were not detected in the course of above-mentioned Michael reactions, under the reaction conditions.

We then screened various electronically and sterically different phosphines such as PPh_3_, Bu_3_P, (*i*Pr)_3_P and PMe_3_. Initial experiments revealed that the catalytic activity of phosphines and phosphine loading (varying from 20% to 50%) efficiently promoted the Michael reaction. With 50 mol% catalyst loading, the reaction rates were approximately 2- to 3-fold faster than the corresponding 20 mol% PPh_3_ catalyzed reactions. A dramatic increase in the efficiency of the reaction came from the usage of less bulkier phosphine, PMe_3_ (20 mol%), which led to the best result (Table 2, entry 1).

**Table 2:** PMe_3_-catalyzed reaction of fluoro(phenylsulfonyl)-substituted methane derivatives with methyl vinyl ketone and ethyl acrylate.

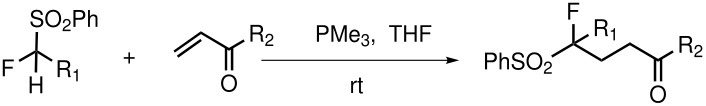					
Entry	R_1_	R_2_	Product	Time (h)	Yield^a^ (%)

1	NO_2_	Me	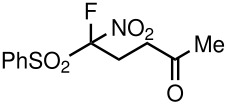 **5a**	40	93
2	CN	Me	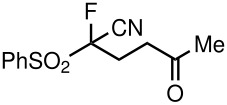 **5b**	22	90
3	CO_2_Et	Me	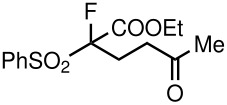 **5c**	24	75
4	PhSO_2_	Me	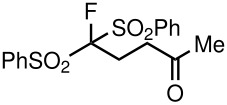 **5d**	17	91
5	NO_2_	OEt	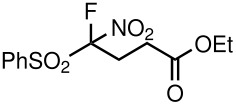 **5e**	72	64
6	CN	OEt	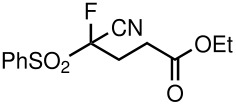 **5f**	72	60
7	CO_2_Et	OEt	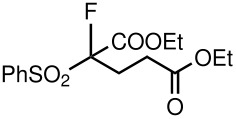 **5g**	116	71
8	PhSO_2_	OEt	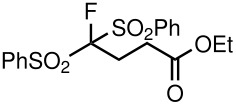 **5h**	67	88

^a^isolated yield

Having established optimal conditions, we then investigated the scope of various substrates in the reaction (Table 2). Methyl vinyl ketone reacted with **3a**, **3b**, **3c**, or **3f** to furnish the corresponding products in good to excellent yields, respectively, except in the case of 1-fluoro-1-(phenylsulfonyl)propane-2-one (**3d**) (Table 2, entries 1–4). Compound **3d** gave complex results due to the reactions of the two types of active acidic α-Hs adjacent to the carbonyl group in the molecule.

When ethyl acrylate was subjected to similar reaction conditions, the yields of the products were 60–88% after prolonged reaction time. Compared to methyl vinyl ketone, the reaction rates were slow due to the somewhat lower reactivity of ethyl acrylate (Table 2, entries 5–8). All products obtained were characterised by ^19^F, ^1^H, ^13^C NMR spectra, as well as HRMS. In addition, base-sensitive functional groups such as cyano, nitro, and ester were well tolerated during the course of the reaction. The failure of (*E*)-pent-3-en-2-one to undergo the Michael reaction under these conditions demonstrates that the reaction is not tolerant of substituents at the terminal position of the double bond due to steric effects.

**Scheme 1:**
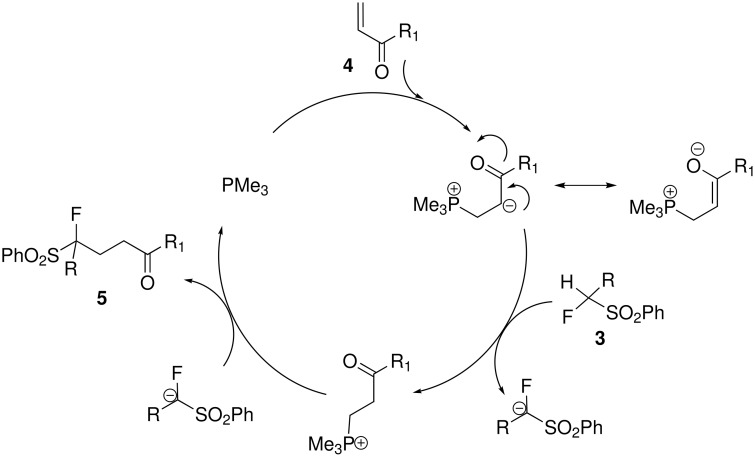
Reaction mechanism for phosphine catalyzed 1,4-addition to α,β-unsaturated compounds.

On the basis of the above mentioned results, a proposed mechanism for the formation of **5a**–**h** is outlined in Scheme 1. Trialkylphosphine catalysed Morita-Baylis-Hillman reaction is well studied by a number of groups [37–39]. Addition of PMe_3_ to alkene **4** generates the dipolar intermediate. The latter abstracts a proton from the α-fluoro-substituted methylene derivative **3**, followed by an intermolecular S_N_2 reaction to furnish the desired product **5** and the release of PMe_3_ (Scheme 1). The mechanism supports the fact that the less steric hindered catalyst PMe_3_ is more efficient than PPh_3_, Bu_3_P or (*i*Pr)_3_P.

In addition, the presence of electron withdrawing groups such as the phenylsulfonyl group can be exploited to generate a carbanion that can act as a "soft" nucleophile [40–41]. The phenylsulfonyl group delocalises the electron density on the fluorinated carbanion center, which makes the resulting nucleophile softer and more suitable for 1,4-addition with Michael acceptors. Hence, we explored the possibility of a base induced Michael addition reaction. Among the various bases and solvent combinations that we explored, the K_2_CO_3_/DMF system was found to be very efficient both in terms of conversions as well as reaction times.

The reactions were carried out at room temperature and the completion observed within 2 h. The reaction was found to be versatile for various α,β-unsaturated compounds such as ketones, esters, nitriles and sulfones (Table 3). In case of α,β-unsaturated aldehydes the reaction was found to be not clean and too many fluorine peaks appeared in the ^19^F NMR even when the reaction was carried out at low temperatures. On the other hand, α,β-unsaturated nitriles underwent a second Michael addition of the product **6h**. Both fluoro(bisphenylsulfonyl)methane and fluoronitro(phenylsulfonyl)methane were added to propynoates under similar conditions. As expected, a mixture of both *cis* and *trans* products were obtained as shown in Table 3 (entries 10–11). Interestingly, in the case of fluoronitro(phenylsulfonyl)methane only the *trans* isomer **6l** was obtained in an appreciable amount while the *cis* product was observed only in traces.

**Table 3:** K_2_CO_3_/DMF catalyzed 1,4-addition to α,β-unsaturated esters, ketones, sulfone, nitriles and propynoates.

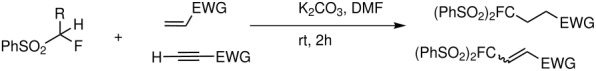				
Entry	R	Substrate	Product (**5**/**6**)	Yield^a^

1	PhSO_2_	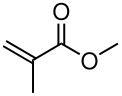	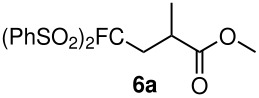	70
2	PhSO_2_	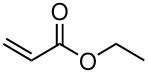	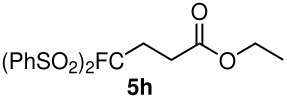	70
3	PhSO_2_	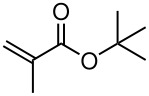	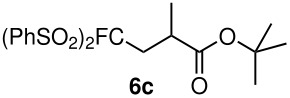	71
4	PhSO_2_	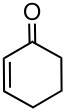	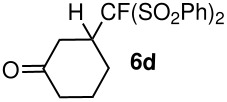	90
5	PhSO_2_	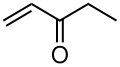	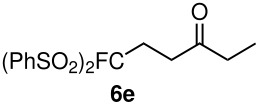	79
6	PhSO_2_	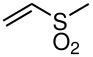	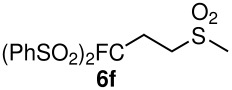	60
7	PhSO_2_	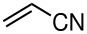	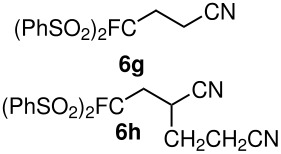	54 (1:2)
8	NO_2_	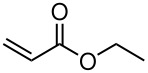	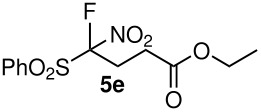	45
9	COOEt	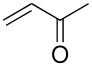	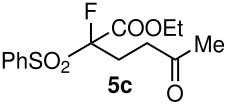	65^b^
10	PhSO_2_	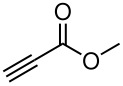	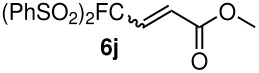	76 (2:3)^c^
11	PhSO_2_	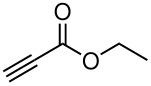	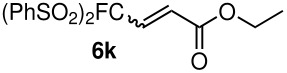	60 (1:1)^c^
12	NO_2_	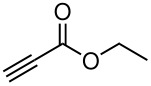	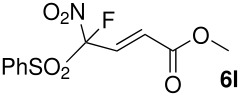	46^d^

^a^isolated yield, ^b^NMR yield (based on ^19^F NMR), ^c^*cis : trans* ratio, ^d^only trace amount of *cis* isomer observed

During our study, we observed that the steric factor affects the addition of the pronucleophile to the Michael acceptor. Substitution at the α-position of the Michael acceptor affects the reactivity of the nucleophile generated by the K_2_CO_3_/DMF system. The reaction of fluoro(bisphenylsulfonyl)methane with methyl crotonate was found to give only 50% conversion based on ^19^F NMR after 36 h and methyl cinnamate did not react at all at room temperature using the K_2_CO_3_/DMF system. These observations are consistent with what was observed in the phosphine case discussed earlier. Attempted reductive desulfonylation on compound **6d** using Mg/CH_3_OH [11] was not selective as simultaneous reduction of the carbonyl group was also observed.

## Conclusion

In summary, a convenient protocol for the preparation of α-substituted fluoro(phenylsulfonyl)methane derivatives has been described and its subsequent use in 1,4-addition to a variety of Michael acceptors has also been demonstrated. Further applications of fluoro(phenylsulfonyl)methane including stereocontrolled synthesis will be reported in due course.

## Supporting Information

File 1 Experimental procedures, full spectroscopic data and spectra.

File 2 Spectra.
