# Supplementary material for: Efficient 1,4-addition of α-substituted fluoro(phenylsulfonyl)methane derivatives to α,β-unsaturated compounds
Source: Beilstein J Org Chem. 2008 May 21;4:17. doi: 10.3762/bjoc.4.17 (PMC2486456; doi:10.3762/bjoc.4.17)
Supplement: File 1 — Experimental procedures, full spectroscopic data and spectra. [file Beilstein_J_Org_Chem-04-17-s001.doc]

**Supporting Information (Part 1)**

**Efficient 1,4-addition of α-substituted fluoro(phenylsulfonyl)methane derivatives to α,β-unsaturated compounds**

*G. K. Surya Prakash,* Xiaoming Zhao, Sujith Chacko, Fang Wang, Habiba Vaghoo and George A. Olah**

*Loker Hydrocarbon Research Institute and Department of Chemistry, University of Southern California, Los Angeles, California 90089-1661*

[*gprakash@usc.edu*](mailto:gprakash@usc.edu)*, olah@usc.edu*

Supporting Information S2-S13

Spectra S14-S34

**Experimental Section**

Unless otherwise mentioned, all other reagents were purchased from commercial sources. Diethyl ether and THF were all distilled under nitrogen over sodium/benzophenone prior to use. Toluene was distilled over sodium. Column chromatography was carried out using silica gel (60-200 mesh).

^1^H, ^13^C and ^19^F NMR spectra were recorded on Varian Mercury 400 NMR spectrometers. ^1^H NMR chemical shifts were determined relative to internal (CH_3_)_4_Si (TMS) at δ 0.0 ppm or to the signal of a residual protonated solvent: CDCl_3_ δ 7.26 ppm. ^13^C NMR chemical shifts were determined relative to internal TMS at δ 0.0 ppm or to the ^13^C signal of solvent: CDCl_3_ δ 77.16 ppm. ^19^F NMR chemical shifts were determined relative to internal CFCl_3_ at δ 0.0 ppm.

**A general procedure for the oxidation of sulfides**

Aqueous hydrogen peroxide (13.5 mL, 160 mmol, 30% wt) was added into a solution of the sulfide (40 mmol) in CH_3_CO_2_H (60 mL) at 0 °C and the reaction mixture was stirred about 2 h. The reaction mixture was allowed to warm to room temperature and stirred overnight. The mixture was poured into ice water (200 mL) and extracted with dichloromethane (200 mL). The organic phase was washed with ice water (200 mL × 3), dried over MgSO_4_, and evaporated. The residue was purified by column chromatography to afford the title product (**2a**–**2e**). The characterization data of (**2a**–**2e**) [1-6], **3a** [7], **3c** [8] and **3d** [9] are consistent with those reported earlier.

**(Nitromethylsulfonyl)benzene** (**2a**):

White solid, 90% yield. ^1^H NMR (CDCl_3_): δ 5.60 (s, 2H), 7.64–7.68 (m, 2H), 7.78–7.82 (m, 1H), 7.96–7.99 (m, 2H).

**2-(Phenylsulfonyl)acetonitrile** (**2b**):

Colorless oil, 76% yield. ^1^H NMR (CDCl_3_): δ 4.07 (s, 2H), 7.65–7.70 (m, 2H), 7.77–7.82 (m, 1H), 8.04–8.06 (m, 2H).

**Ethyl 2-(phenylsulfonyl)acetate** (**2c**):

Colorless oil, in 83% yield. ^1^H NMR (CDCl_3_): δ 1.29 (t, *J* = 7.2 Hz, 3H), 4.26–4.42 (m, 2H), 7.59–7.64 (m, 2H), 7.73–7.78 (m, 1H), 7.93–7.95 (m, 2H).

**1-(Phenylsulfonyl)propan-2-one** (**2d**):

Colorless oil, in 70% yield. ^1^H NMR (CDCl_3_): δ 2.42 (s, 3H), 4.16 (s, 2H), 7.57–7.61 (m, 2H), 7.68–7.72 (m, 1H), 7.88–7.91 (m, 2H).

**(Allylsulfonyl)benzene** (**2e**):

Colorless oil, in 91% yield. ^1^H NMR (CDCl_3_): δ 3.80–3.82 (m, 2H), 5.12–5.13 (m, 1H), 5.16–5.17 (m, 1H), 5.74–5.84 (m, 1H), 7.53–7.58 (m, 2H), 7.63–7.86 (m, 1H), 7.87–7.89 (m, 2H).

**Improved procedure for the monofluorination with Selectfluor**: To a slurry of NaH (6.75 mmol) in THF (15 mL) was added a solution of 1-substituted (phenylsulfonyl)methane (6.75 mmol) in THF (10 mL) at 0 °C under argon. After stirring for 2 h, a solution of Selectfluor (6.75 mmol) in anhydrous DMF (15 mL) was added dropwise to the above-mentioned mixture at 0 °C and the reaction mixture was stirred overnight, and monitored by TLC. After completion of the reaction, CH_2_Cl_2_ (20 mL) was added and the mixture was washed with water, brine, then the organic phase was dried with MgSO_4_ and filtered. The filtrate was evaporated, and the residue was purified by column chromatography to give the title product in good yield.

**[Fluoro(nitro)methylsulfonyl]benzene (3a):**

White solid isolated in 62% yield. ^1^H NMR (CDCl_3_): δ 6.44 (d, *J* = 48.4 Hz, 1H), 7.66−7.70 (m, 2H), 7.83−7.87 (m, 1H), 7.94−7.96 (m, 2H). ^13^C NMR (CDCl_3_): δ 111.81 (d, *J* = 283.0 Hz), 130.06, 130.72, 131.43, 136.84. ^19^F NMR (CDCl_3_): δ −142.16 (d, *J* = 48.4 Hz, 1F).

**2-Fluoro-2-(phenylsulfonyl)acetonitrile (3b):**

Colorless oil isolated in 45% yield. ^1^H NMR (CDCl_3_): δ 5.69 (d, *J* = 46.8 Hz, 1H), 7.68–7.73 (m, 2H), 7.84–7.88 (m, 1H), 8.04–8.07 (m, 2H). ^13^C NMR (CDCl_3_): δ 88.11 (d, *J* = 232.3 Hz), 109.87 (d, *J* = 29.5 Hz), 130.00, 130.69, 132.25, 136.59. ^19^F NMR (CDCl_3_): δ −179.23 (d, *J* = 47.8 Hz, 1F). HRMS Calcd for C_8_H_6_FNO_2_S: 199.0103; Found: 199.0107.

**Ethyl 2-fluoro-2-(phenylsulfonyl)acetate (3c):**

Colorless oil isolated in 56% yield. ^1^H NMR (CDCl_3_): δ 1.29 (t, *J* = 7.2 Hz, 3H), 4.29 (q, 2H), 5.57 (d, *J* = 48.0 Hz, 1H), 7.59–7.64 (m, 2H), 7.73–7.78 (m, 1H), 7.93–7.96 (m, 2H). ^13^C NMR (CDCl_3_): δ 14.03, 63.64, 97.39 (d, *J* = 232.5 Hz), 129.54, 129.93, 134.65, 135.46, 161.11 (d, *J* = 22.9 Hz). ^19^F NMR (CDCl_3_): δ −180.91 (d, *J* = 48.0 Hz, 1F).

**1-Fluoro-1-(phenylsulfonyl)propan-2-one (3d):**

White solid isolated in 61% yield. ^1^H NMR (CDCl_3_): δ 2.37 (d, *J* = 3.6 Hz, 3H), 5.46 (d, *J* = 49.2 Hz, 1H), 7.61–7.65 (m, 2H), 7.75–7.77 (m, 1H), 7.92–7.95 (m, 2H). ^13^C NMR (CDCl_3_): δ 27.65, 101.51 (d, *J* = 874.8 Hz), 129.68, 129.74, 134.95, 135.51, 195.71 (d, *J* = 81.7 Hz). ^19^F NMR (CDCl_3_): δ −113.56 (tq, *J* = 49.2 Hz, 3.6 Hz, 1F).

**General procedure**:

To a solution of fluorine-substituted active methylene derivative (0.2 mmol, 1 equivalent) and methyl vinyl ketone (0.6 mmol, 3 equivalents) in THF (0.5 mL) was added trimethylphosphine (0.04 mmol, 1.0 M in THF) dropwise by syringe at room temperature under argon, and the reaction was monitored by TLC. After completion of the reaction, the solvents were removed under vacuum. The crude products were purified by column chromatography (hexane/ ethyl acetate = 3/1) to afford the products in good to excellent yield.

**5-Fluoro-5-nitro-5-(phenylsulfonyl)pentan-2-one (5a):**

White solid isolated in 93% yield. ^1^H NMR (CDCl_3_): δ 2.18 (s, 3H), 2.50–2.56 (m, 1H), 2.70–2.78 (m, 1H), 2.88–2.92 (m, 1H), 2.98–3.09 (m, 1H), 7.62–7.66 (m, 2H), 7.79–7.83 (m, 1H), 7.91–7.94 (m, 2H). ^13^C NMR (CDCl_3_): δ 25.56 (d, *J* = 17.9 Hz), 27.19, 30.01, 35.53 (d, *J* = 2.2 Hz), 123.63 (d, *J*=282.4), 125.04, 129.91, 131.09, 131.33, 136.55, 203.79. ^19^F NMR (CDCl_3_): δ −125.90, −125.96 (dd, *J*_1_ = 28.6 Hz, 10.5 Hz, 1F). HRMS: (FAB) calcd for C_11_H_13_FNO_5_S 290.0499 (M+1) found: m/z 290.0500.

**2-Fluoro-5-oxo-2-(phenylsulfonyl)hexanenitrile (5b):**

White solid isolated in 90% yield. ^1^H NMR (CDCl_3_): δ 2.23 (s, 3H), 2.66–2.75 (m, 1H), 2.85–2.92 (m, 1H), 7.65–7.69 (m, 2H), 7.81–7.85 (m, 1H), 8.02–8.04 (m, 2H). ^13^C NMR (CDCl_3_): δ 25.84 (d, *J* = 21.4 Hz), 30.00, 36.82 (d, *J* = 2.4 Hz), 99.20 (d, *J* = 228.8 Hz), 112.24 (d, *J* = 33.1 Hz), 129.75, 131.14, 132.04, 136.32, 204.03. ^19^F NMR (CDCl_3_): δ −148.50 (dd, *J*= 27.1 Hz, 16.9 Hz, 1F). HRMS: (FAB) calcd for C_12_H_13_FNO_3_S 270.0600 (M+1) found: m/z 270.0612.

**Ethyl 2-fluoro-5-oxo-2-(phenylsulfonyl)hexanoate (5c):**

White solid isolated in 75% yield. ^1^H NMR (CDCl_3_): δ 1.20 (t, *J* = 7.2 Hz, 3H), 2.14 (s, 3H), 2.49–2.56 (m, 2H), 2.67–2.78 (m, 2H), 4.15–4.23 (m, 2H), 7.56–7.60 (m, 2H), 7.70–7.74 (m, 1H), 7.89–7.92 (m, 2H). ^13^C NMR (CDCl_3_): δ 13.93, 25.28 (d, *J* = 19.9 Hz), 29.92, 36.48 (d, *J* = 3.2 Hz), 63.58, 106.26 (d, *J* = 233.3 Hz), 129.30, 130.45, 134.13, 135.24, 163.63 (d, *J* = 24.8 Hz), 205.27. ^19^F NMR (CDCl_3_): δ −158.34 (m, 1F). HRMS: (FAB) calcd for C_14_H_18_FNO_5_S 317.0859 (M+1) found: m/z 317.0861.

**5-Fluoro-5,5-bis(phenylsulfonyl)pentan-2-one (5d):**

White solid isolated in 91% yield. ^1^H NMR (CDCl_3_): δ 2.17 (s, 3H), 2.56–2.64 (m, 2H), 3.05–3.09 (m, 2H), 7.55–7.59 (m, 4H), 7.72–7.76 (m, 2H), 7.91–7.93 (m, 4H). ^13^C NMR (CDCl_3_): δ 24.81 (d, *J* = 18.9 Hz), 29.95, 36.51 (d, *J* = 6.2 Hz), 115.07 (d, *J* = 264.4 Hz), 129.20, 130.99, 134.89, 135.52. ^19^F NMR (CDCl_3_): δ −140.70 (t, *J* = 15.4 Hz, 1F). HRMS: (FAB) calcd for C_17_H_18_FO_5_S_2_ 385.0580 (M+1) found: m/z 385.0565.

**Ethyl 4-fluoro-4-nitro-4-(phenylsulfonyl)butanoate (5e):**

White solid isolated in 64% yield. ^1^H NMR (CDCl_3_): δ 1.26 (t, *J* = 7.2 Hz, 3H), 2.31–2.39 (m, 1H), 2.50–2.58 (m, 1H), 2.87–2.95 (m, 1H), 3.04–3.20 (m, 1H), 4.11 (q, *J* = 7.2 Hz, 2H), 7.58–7.62 (m, 2H), 7.76–7.80 (m, 1H), 7.88–7.90 (m, 2H). ^13^C NMR (CDCl_3_): δ 14.37, 26.70 (d, *J* = 18.2 Hz), 27.04 (d, *J* = 3.8 Hz), 61.73, 123.43 (d, *J* = 282.8 Hz), 130.04, 131.22, 131.27, 136.73, 170.35. ^19^F NMR (CDCl_3_): δ −126.70 (dd, *J*= 30.1 Hz, 9.8 Hz, 1F). HRMS: (FAB) calcd for C_12_H_15_FNO_6_S 320.0604 (M+1) found: m/z 320.0612.

**Ethyl 4-cyano-4-fluoro-4-(phenylsulfonyl)butanoate (5f)**

White solid isolated in 60% yield. ^1^H NMR (CDCl_3_): δ 1.30 (t, *J* = 7.2 Hz, 3H), 2.70–2.83 (m, 4H), 4.17–4.22 (m, *J* = 7.2 Hz, 2H), 7.66–7.70 (m, 2H), 7.81–7.86 (m, 1H), 8.03–8.06 (m, 2H). ^13^C NMR (CDCl_3_): δ 14.32, 27.59 (d, *J* = 18.9 Hz), 28.33 (d, *J* = 3.0 Hz), 61.59, 99.04 (d, *J* = 228.8 Hz), 112.15 (d, *J* = 33.2 Hz), 129.86, 131.28, 132.08, 136.44, 170.49. ^19^F NMR (CDCl_3_): δ −149.63 (m, 1F). HRMS: (FAB) calcd for C_13_H_15_FNO_4_S 300.0706 (M+1) found: m/z 300.0719.

**Diethyl 2-fluoro-2-(phenylsulfonyl)pentanedioate (5g):**

White solid isolated in 71% yield. ^1^H NMR (CDCl_3_): δ 1.18–1.25 (m, 6H), 2.32–2.38 (m, 1H), 2.55–2.59 (m, 2H), 2.76–2.84 (m, 1H), 4.06–4.21 (m, 4H), 7.54–7.58 (m, 2H), 7.68–7.72 (m, 1H), 7.87–7.90 (m, 2H). ^13^C NMR (CDCl_3_): δ 14.15, 14.41, 26.63 (d, *J* = 20.2 Hz), 28.05 (d, *J* = 3.4 Hz), 61.31, 63.82, 106.38 (d, *J* = 234.5 Hz), 129.51, 130.67, 134.23, 135.47, 163.61 (d, *J* = 25.5 Hz), 171.40. ^19^F NMR (CDCl_3_): δ −159.13 (dd, *J*= 31.2 Hz, 10.5 Hz, 1F). HRMS: (FAB) calcd for C_15_H_20_FNO_6_S 347.0965 (M+1) found: m/z 347.0962.

**Ethyl 4-fluoro-4,4-bis(phenylsulfonyl)butanoate (5h):**

White solid isolated in 88% yield. ^1^H NMR (CDCl_3_): δ 1.20 (t, *J* = 7.2 Hz, 3H), 2.62–2.71 (m, 2H), 2.78–2.83 (m, 2H), 4.04–4.09 (m, 2H), 7.51–7.56 (m, 2H), 7.68–7.72 (m, 1H), 7.88–7.90 (m, 2H). ^13^C NMR (CDCl_3_): δ 14.22, 26.01 (d, *J* = 19.4 Hz), 27.69 (d, *J* = 7.7 Hz), 61.06, 114.75 (d, *J* = 266.1 Hz), 129.21, 130.96, 134.85, 135.52, 171.43. ^19^F NMR (CDCl_3_): δ −142.91 (t, *J* = 15.8 Hz, 1F). HRMS: (FAB) calcd for C_18_H_20_FO_6_S_2_ 415.0685 (M+1) found: m/z 415.0673.

**General procedure**:

To a solution of fluorine-substituted active methylene derivative (0.16 mmol, 1 equivalent) and α,β-unsaturated compound (0.16 mmol, 1 equivalent) in DMF (2 mL) was added potassium carbonate (0.36 mmol, 0.050 g) at room temperature and the reaction was monitored by ^19^F NMR. Aqueous work up followed by extraction with dichloromethane (3 x 15 mL) afforded the crude product. The pure product was obtained by column chromatography.

**4,4-Bis(benzenesulfonyl)-4-fluoro-2-methyl-butyric acid methyl ester (6a)**

Colorless oil isolated in 70% yield. ^1^H NMR (CDCl_3_): δ 1.18 (d, *J* = 6.8 Hz, 3H), 2.44–2.58 (m, 1H), 2.50–2.58 (m, 1H), 2.92 (dd, *J* = 29.4 Hz, 0.5 Hz, 1H), 2.96–3.11 (m, 2H), 3.51 (s, 3H), 7.55–7.61 (m, 4H), 7.71–7.77 (m, 2H), 7.87–7.99 (m, 4H). ^13^C NMR (CDCl_3_): δ 18.99, 32.84 (d, *J* = 17.5 Hz), 34.74 (d, *J* = 5.8 Hz), 51.95, 114.80 (d, *J* = 268.6 Hz), 128.96, 129.01, 130.93, 130.95, 134.41, 134.77, 135.28, 135.32, 175.07 ^19^F NMR (CDCl_3_): δ −146.50 (dd, *J* = 22.8 Hz, 10.5 Hz, 1F) HRMS: (FAB) calcd for C_18_H_19_FO_6_S_2_ 415.0685 (M+1) found: m/z 415.0668.

**4,4-Bis(benzenesulfonyl)-4-fluoro-2-methyl-butyric acid tert-butyl ester (6c)**

White solid isolated in 70% yield. ^1^H NMR (CDCl_3_): δ 1.13 (d, *J* = 7.1Hz, 3H), 1.34 (s, 9H), 2.43 (ddd, *J* =23.5 Hz, 15.8 Hz, 3.9 Hz, 1H), 2.80–2.90 (m, 1H), 2.91–3.02 (m, 1H), 7.54–7.62 (m, 4H), 7.70–7.77 (m, 2H), 7.88–8.00 (m, 4H). ^13^C NMR (CDCl_3_): δ 19.03, 27.75 (d, *J* = 11.9 Hz), 32.81 (d, *J* = 17.3 Hz), 35.76 (d, *J* = 5.7 Hz), 80.52, 115.11 (d, *J* = 268.4 Hz), 128.96, 129.01, 130.96, 134.53, 134.95, 135.23, 135.26, 173.93. ^19^F NMR (CDCl_3_): δ −146.17 (dd, *J* = 10.7 Hz, 23.6 Hz, 1F) HRMS: (FAB) calcd for C_21_H_25_FO_6_S_2_ 457.1155 (M+1) found: m/z 457.1147.

**3-[Bis(benzenesulfonyl)-fluoro-methyl]-cyclohexanone (6d)**

White solid isolated in 90% yield. ^1^H NMR (CDCl_3_): δ 1.40–1.56 (m, 1H), 2.15–2.25 (m, 1H), 2.30–2.47 (m, 3H), 2.52–2.80 (m, 3H), 3.03–3.13 (m, 1H), 7.48–7.57 (m, 4H), 7.68–7.75 (m, 2H), 7.77–7.83 (m, 2H), 7.85–7.91 (m, 2H) ^13^C NMR (CDCl_3_): δ 25.10, 25.15, 41.07, 41.36 (d, *J* = 6.9 Hz), 42.67 (d, *J* = 17.2 Hz), 114.41 (d, *J* = 266.4 Hz), 114.45, 128.83, 129.09, 130.58, 130.59, 130.76, 130.78, 134.87, 135.20, 135.34, 135.77, 207.82. ^19^F NMR (CDCl_3_): δ −136.36 (s, 1F) HRMS: (FAB) calcd for C_19_H_19_FO_5_S_2_ 411.0736 (M+1) found: m/z 411.0740.

**6,6-Bis(benzenesulfonyl)-6-fluoro-hexan-3-one (6e)**

White solid isolated in 79% yield. ^1^H NMR (CDCl_3_): δ.1.04 (d, *J* = 7.4 Hz, 3H), 2.46 (q, *J* = 7.3 Hz, 2H), 2.58–2.68 (m, 2H), 3.00–3.08 (m, 2H), 7.55–7.62 (m, 4H), 7.70–7.77 (m, 2H), 7.90–7.95 (m, 4H) ^13^C NMR (CDCl_3_): δ 24.75 (d, *J* = 18.4 Hz), 35.00, 35.05, 35.82, 115.03 (d, *J* = 264.5 Hz), 129.06, 130.83, 130.85, 134.79, 135.37, 207.99. ^19^F NMR (CDCl_3_): δ −140.74 (t, *J* = 15.9 Hz, 1F) HRMS: (FAB) calcd for C_18_H_19_FO_5_S_2_ 399.0736 (M+1) found: m/z 399.0742.

**3,3-Bis(benzenesulfonyl)-3-fluoro-1-(methylsulfonyl)propane (6f)**

White solid isolated in 60% yield. ^1^H NMR (CDCl_3_): δ.2.77–2.87 (m, 2H), 2.99 (s, 3H), 3.60–3.67 (m, 2H), 7.57–7.63 (m, 4H), 7.74–7.80 (m, 2H), 7.91–7.96 (m, 4H) ^13^C NMR (CDCl_3_): δ 24.01(d, *J* = 20.7 Hz), 40.99, 48.12 (d, *J* = 6.9 Hz), 113.11 (d, *J* = 266.1Hz), 129.29, 130.94, 134.01, 135.82 ^19^F NMR (CDCl_3_): δ −140.35 (t, *J* = 14.0 Hz, 1F) HRMS: (FAB) calcd for C_16_H_17_FO_6_S_3_ 421.0249 (M+1) found: m/z 421.0262.

**4,4-Bis(benzenesulfonyl)-4-fluoro-butyronitrile (6g)**

White solid. ^1^H NMR (CDCl_3_): δ 2.70–2.79 (m, 2H), 2.91–2.97 (m, 2H), 7.56–7.63 (m, 4H), 7.80–7.74 (m, 2H), 7.88–7.93 (m, 4H). ^13^C NMR (CDCl_3_): δ 12.07 (d, *J =* 9.2 Hz), 26.94 (d, *J* = 18.4 Hz), 113.00 (d, *J* = 267.6 Hz), 117.561, 129.34, 130.83, 134.10, 135.82. ^19^F NMR (CDCl_3_): δ −145.04 (t, *J* = 15.5 Hz, 1F) HRMS: (FAB) calcd for C_16_H_14_FNO_4_S_2_ 368.0426 (M+1) found: m/z 368.0445.

**2-[2,2-Bis(benzenesulfonyl)-2-fluoroethyl]-pentanedinitrile (6h)**

White solid. ^1^H NMR (CDCl_3_): δ 1.90**–**2.20 (m, 2H), 2.43–2.68 (m, 2H), 2.76–2.88 (m, 2H), 3.32–3.42 (m, 1H), 7.55–7.64 (m, 4H), 7.74–7.82 (m, 2H), 7.84–7.88 (m, 2H), 7.93–7.97 (m, 2H). ^13^C NMR (CDCl_3_): δ 15.09, 25.95 (d, *J =* 5.4 Hz), 28.81, 32.13 (d, *J =* 17.6 Hz), 112.95 (d, *J =* 269.9 Hz), 117.37, 118.56, 129.32, 129.51, 130.80, 131.01, 133.35, 133.95, 135.95. ^19^F NMR (CDCl_3_): δ −146.55 (dd, *J* = 19.5 Hz, 12.9 Hz, 1F) HRMS: (FAB) calcd for C_19_H_17_FN_2_O_4_S_2_ 421.0692 (M+1) found: m/z 421.0688.

**4,4-Bis(benzenesulfonyl)-4-fluoro-but-2-enoic acid methyl ester (6j)**

*cis*

^1^H NMR (CDCl_3_): δ 3.75 (s, 3H), 5.97 (d, *J* = 15.7 Hz, 1H), 7.27 (dd, *J* = 23.1 Hz, 15.7 Hz, 1H), 7.55–7.61 (m, 4H), 7.72–7.78 (m, 2H), 7.91–7.96 (m, 4H). ^13^C NMR (CDCl_3_): δ 52.43, 112.86 (d, *J =* 269.1 Hz), 129.12, 129.77 (d, *J =* 9.2 Hz), 130.59 (d, *J =* 14.6 Hz), 130.89, 134.40, 135.79. ^19^F NMR (CDCl_3_): δ −150.93 (d, *J* = 23.1 Hz, 1F) HRMS: (FAB) calcd for C_17_H_15_FO_6_S_2_ 399.0372 (M+1) found: m/z 399.0379.

*trans*

^1^H NMR (CDCl_3_): δ 3.42 (s, 3H), 6.18 (dd, *J* = 12.9 Hz, 1.3 Hz, 1H), 6.44 (dd, *J* = 28.5 Hz, 12.9 Hz, 1H), 7.56–7.62 (m, 4H), 7.73–7.78 (m, 2H), 7.93–7.98 (m, 4H).  ^13^C NMR (CDCl_3_): δ 51.96, 113.53 (d, *J =* 273.0 Hz), 121.26 (d, *J =* 13.80 Hz), 128.98, 130.78 (d, *J =* 3.07 Hz), 131.06, 134.48, 135.54, 164.13. ^19^F NMR (CDCl_3_): δ −150.60 (d, *J* = 28.5 Hz, 1F) HRMS: (FAB) calcd for C_17_H_15_FO_6_S_2_ 399.0372 (M+1) found: m/z 399.0379.

**4,4-Bis(benzenesulfonyl)-4-fluoro-but-2-enoic acid ethyl ester (6k)**

*cis*

White solid. ^1^H NMR (CDCl_3_): δ 1.28 (t, *J* = 7.1 Hz, 3H), 4.19 (q, *J* = 7.1 Hz, 2H), 5.96 (dd, *J =* 15.7 Hz, 0.4 Hz, 1H), 7.25 (dd, *J* = 23.1 Hz, 15.7 Hz, 1H), 7.55–7.62 (m, 4H), 7.72–7.78 (m, 2H), 7.91–7.96 (m, 4H). ^13^C NMR (CDCl_3_) : δ 14.00, 61.44, 112.80 (d, *J =* 269.1 Hz), 129.06, 130.09 (d, *J =* 9.1 Hz), 130.21 (d, *J =* 3.2 Hz), 130.79, 134.31, 135.75, 163.23. ^19^F NMR (CDCl_3_): δ −150.90 (d, *J* = 23.1 Hz, 1F) HRMS: (FAB) calcd for C_18_H_17_FO_6_S_2_ 413.0529 (M+1) found: m/z 413.0543.

*trans*

White solid. ^1^H NMR (CDCl_3_): δ 1.07 (dt, *J* = 7.1 Hz, 0.4 Hz, 3H), 3.91 (q, *J* = 7.1Hz, 2H), 6.17 (dd, *J* = 12.9 Hz, 1.3 Hz, 1H), 6.41 (dd, *J* = 28.7 Hz, 13.0 Hz, 1H), 7.56–7.62 (m, 4H), 7.73–7.78 (m, 2H), 7.94–7.98 (m, 4H).  ^13^C NMR (CDCl_3_): δ 13.85, 61.08, 113.63 (d, *J* = 274.0 Hz), 120.68 (d, *J* = 13.8 Hz), 128.97, 130.98, 130.99, 131.26 (d, *J* = 2.9 Hz), 134.55, 135.50, 163.69. ^19^F NMR (CDCl_3_): δ −150.32 (d, *J* = 28.7 Hz, 1F) HRMS: (FAB) calcd for C_18_H_17_FO_6_S_2_ 413.0529 (M+1) found: m/z 413.0543.

**4-(Benzenesulfonyl)-4-fluoro-4-nitro-but-2-enoic acid ethyl ester (6l)**

White solid. ^1^H NMR (CDCl_3_): δ 1.23 (t, *J =* 7.1 Hz), 4.16 (q , *J =* 7.2 Hz), 6.50 (dd, *J =* 12.4, 2.4 Hz, 1H), 6.79 (dd, *J =* 18.7, 12.3 Hz, 1H), 7.63–7.69 (m, 2H), 7.81–7.87 (m, 1H), 7.92–7.97 (m, 2H). ^13^C NMR (CDCl_3_): δ 13.78, 61.94, 123.83 (d, *J =* 15.3 Hz), 129.68, 131.17, 131.50 (d, *J =* 288.3 Hz), 136.56, 163.12. ^19^F NMR (CDCl_3_): δ −119.05 (d, *J* = 18.3 Hz, 1F) HRMS: (FAB) calcd for C_12_H_12_FNO_6_S 318.0447 (M+1) found: m/z 318.0445.

**References**

1. Wade, P. A.; Hinney, H. R.; Amin, N. V.; Vail, P. D.; Morrow, S. D.; Hardinger, S. A.; Saft, M. S. *J. Org. Chem.* **1981,** *46,* 765–770. doi:10.1021/jo00317a023
2. Truce, W. E.; Klingler, T. C.; Paar, J. E.; Feuer, H.; Wu, D. K. *J. Org. Chem.* **1969,** *34,* 3104–3107. doi:10.1021/jo01262a068
3. Walker, D. *J. Org. Chem.* **1966,** *31,* 835–837. doi:10.1021/jo01341a045
4. Doyle, K. J.; Moody, C. J. *Tetrahedron* **1994,** *50,* 3761–3772. doi:10.1016/S0040-4020(01)90396-5
5. Ohta, H.; Kato, Y.; Tsuchihashi, G. *J. Org. Chem.* **1987,** *52,* 2735–2739. doi:10.1021/jo00389a018
6. Sheldrake, H. M.; Wallace, T. W. *Tetrahedron Lett.* **2007,** *48,* 4407–4411. doi:10.1016/j.tetlet.2007.04.099
7. Prakash, G. K. S.; Chacko, S.; Alconcel, S.; Stewart, T.; Mathew, T.; Olah, G. A. *Angew. Chem., Int. Ed.* **2007,** *46,* 4933–4936. doi:10.1002/anie.200700834
8. Takeuchi, Y.; Asahina, M.; Hori, K.; Koizumi, T. *J. Chem. Soc., Perkin Trans. 1* **1988,** 1149–1153. doi:10.1039/P19880001149
9. Takeuchi, Y.; Ogura, H.; Kanada, A.; Koizumi, T. *J. Org. Chem.* **1992,** *57,* 2196–2199. doi:10.1021/jo00033a058
