# Supplementary material for: Efficient 1,4-addition of α-substituted fluoro(phenylsulfonyl)methane derivatives to α,β-unsaturated compounds
Source: Beilstein J Org Chem. 2008 May 21;4:17. doi: 10.3762/bjoc.4.17 (PMC2486456; doi:10.3762/bjoc.4.17)

**Supporting Information (Part 2)**

**Efficient 1,4-addition of α-substituted fluoro(phenylsulfonyl)methane derivatives to α,β-unsaturated compounds**

G. K. Surya Prakash,* Xiaoming Zhao, Sujith Chacko, Fang Wang, Habiba Vaghoo and George A. Olah*

*Loker Hydrocarbon Research Institute and Department of Chemistry, University of Southern California, Los Angeles, California 90089-1661*

[*gprakash@usc.edu*](mailto:gprakash@usc.edu)*, olah@usc.edu*

Spectra S2-S21


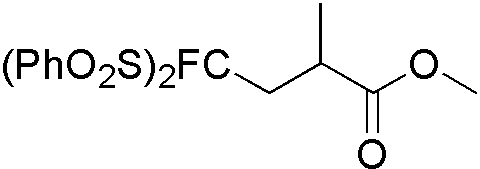


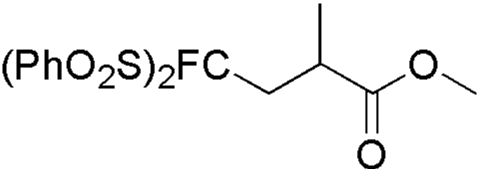


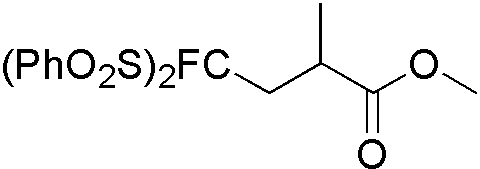

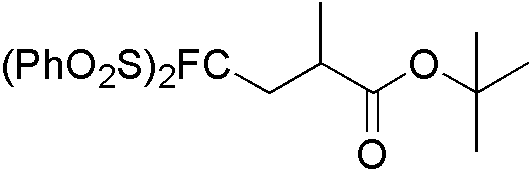


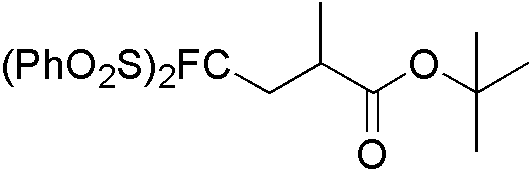


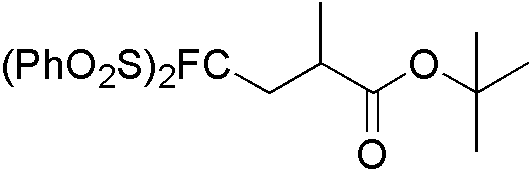


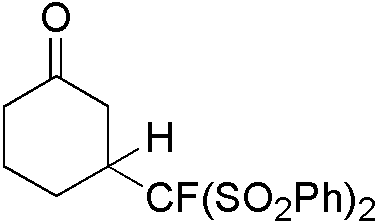


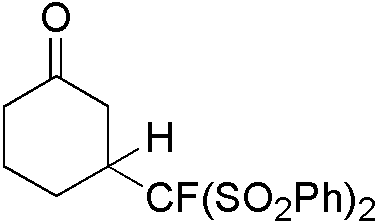


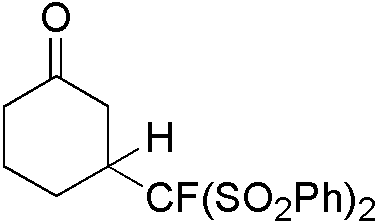


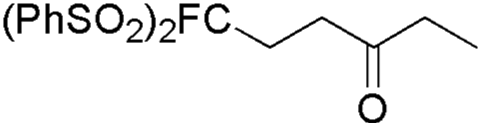


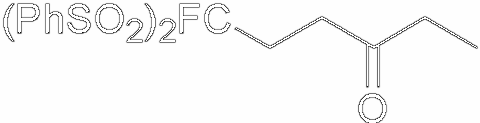


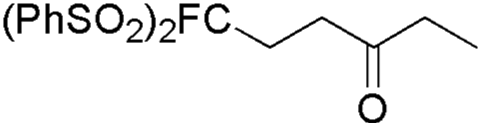


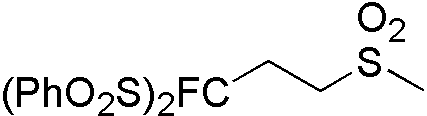


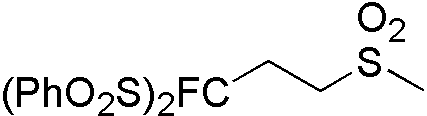


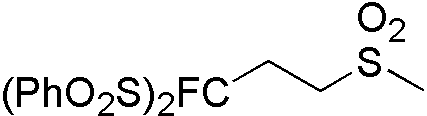


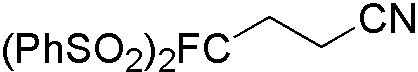


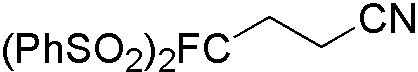


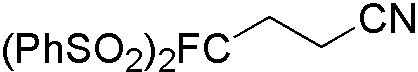


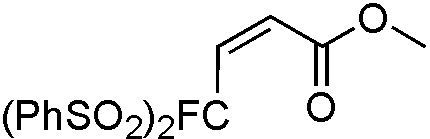


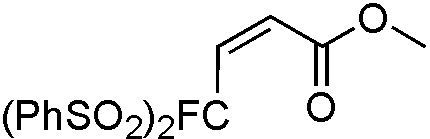


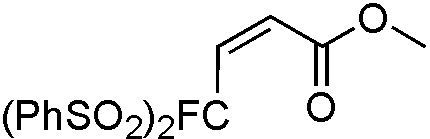


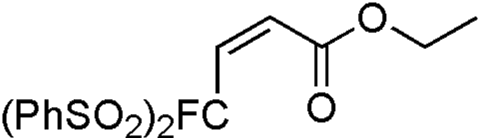


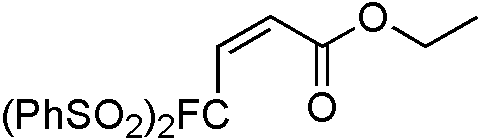


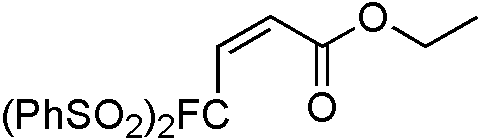


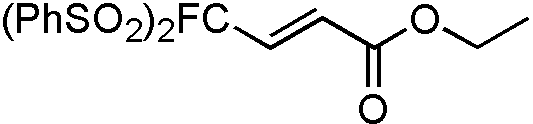


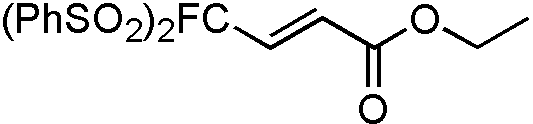


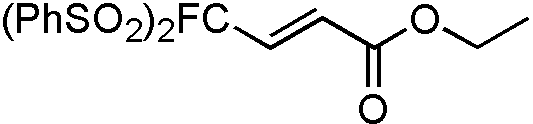


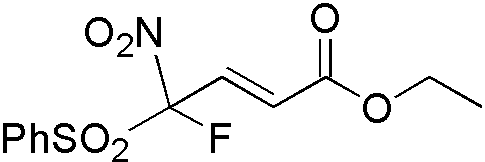


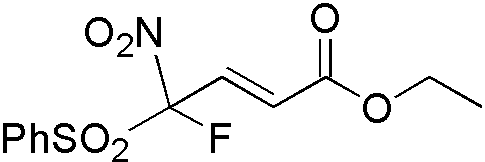


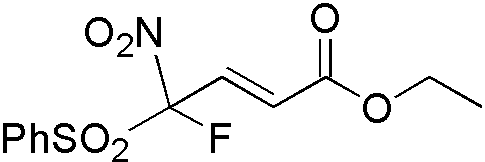

Supplement: File 2 — Spectra. [file Beilstein_J_Org_Chem-04-17-s002.doc]
